# S100A8 and S100A9 Are Associated with Colorectal Carcinoma Progression and Contribute to Colorectal Carcinoma Cell Survival and Migration via Wnt/β-Catenin Pathway

**DOI:** 10.1371/journal.pone.0062092

**Published:** 2013-04-26

**Authors:** Liang Duan, Rui Wu, Liwei Ye, Haiyan Wang, Xia Yang, Yunyuan Zhang, Xian Chen, Guowei Zuo, Yan Zhang, Yaguang Weng, Jinyong Luo, Min Tang, Qiong Shi, Tongchuan He, Lan Zhou

**Affiliations:** 1 Key Laboratory of Diagnostic Medicine designated by the Chinese Ministry of Education, Chongqing Medical University, Chongqing, China; 2 Molecular Oncology Laboratory, Department of Surgery, The University of Chicago Medical Center, Chicago, Illinois, United States of America; Rush University Medical Center, United States of America

## Abstract

**Background and Objective:**

S100A8 and S100A9, two members of the S100 protein family, have been reported in association with the tumor cell differentiation and tumor progression. Previous study has showed that their expression in stromal cells of colorectal carcinoma (CRC) is associated with tumor size. Here, we investigated the clinical significances of S100A8 and S100A9 in tumor cells of CRC and their underlying molecular mechanisms.

**Methods:**

Expression of S100A8 and S100A9 in colorectal carcinoma and matching distal normal tissues were measured by reverse transcriptase polymerase chain reaction (RT-PCR), immunohistochemistry and western blot. CRC cell lines treated with the recombinant S100A8 and S100A9 proteins were used to analyze the roles and molecular mechanisms of the two proteins in CRC *in vitro*.

**Results:**

S100A8 and S100A9 were elevated in more than 50% of CRC tissues and their expression in tumor cells was associated with differentiation, Dukes stage and lymph node metastasis. The CRC cell lines treatment with recombinant S100A8 and S100A9 proteins promoted the viability and migration of CRC cells. Furthermore, the two recombinant proteins also resulted in the increased levels of β-catenin and its target genes c-myc and *MMP7*. β-catenin over-expression in CRC cells by Adβ-catenin increased cell viability and migration. β-catenin knock-down by Adsiβ-catenin reduced cell viability and migration. Furthermore, β-catenin knockdown also partially abolished the promotive effects of recombinant S100A8 and S100A9 proteins on the viability and migration of CRC cells.

**Conclusions:**

Our work demonstrated that S100A8 and S100A9 are linked to the CRC progression, and one of the underlying molecular mechanisms is that extracellular S100A8 and S100A9 proteins contribute to colorectal carcinoma cell survival and migration via Wnt/β-catenin pathway.

## Introduction

Colorectal carcinoma (CRC) is the third leading cause of cancer-associated death worldwide. The CRC incidence rate has been shown to a dropping over these years due to enhancing CRC screening and removing precancerous polyps, but its high recurrence and poor survival rate remain to post a risk to human health [1]–[2]. In recent years, understanding the cancer-specific genes and potential molecular mechanisms during the carcinogenesis and progression of CRC provides an opportunity for the development of targeted therapy for the treatment of CRC.

S100A8 and S100A9, two members of the S100 family of calcium-binding proteins, have been originally detected in myeloid cells and known to play roles in innate immune system [3]–[5]. Recently, the over-expression of S100A8 and S100A9 has been observed in many tumor cells, and is associated with poor cell differentiation in some cancers of glandular cell origin [6]–[9]. Furthermore, elevated expression of S100A8 and S100A9 has been firstly found in CRC by using two-dimensional gel electrophoresis [10]. Then it is reported that expression of S100A8 or S100A9 in stromal cells of CRC is associated with larger-sized tumors [11]. Nevertheless, little research has been done on their expression in tumor cells of CRC and their correlation with CRC progression.

S100A8 and S100A9 proteins have both intracellular and extracellular functions. Intracellular S100A8 and S100A9 are involved in the calcium sensing, activation of NADPH oxidase and arachidonic acid transport [12]–[14]. Extracellular S100A8/A9 heterodimer (25–250 µg/ml) formed by the two calcium-binding proteins S100A8 and S100A9 exerts apoptosis-inducing activity in various cells via suppression of intracellular zinc or interaction with receptor, but in lower doses (<25 µg/ml), either their heterodimer or each alone promotes the viability or migration of tumor cells, vascular endothelial cells and inflammatory cells, which is involved in the binding with receptor for advanced glycation end products (RAGE) through mitogen-activated protein kinase (MAPK) pathway and NF-κB activation [15]–[24]. However, RAGE siRNA does not completely inhibit the cell prolifetation induced by S100A8 or S100A9 [18]. Therefore, the other potential mechanisms responsible for the effect of S100A8 or S100A9 on cell proliferation and migration still need to be investigated.

Wnt/β-catenin pathway plays a critical role in CRC development [25]. The majority of CRC harbor mutations of adenomatous polyposis coli (APC) gene or β-catenin gene, resulting in Wnt-independent cytoplasmic and nuclear β-catenin accumulation [26]–[27]. Furthermore, Wnt/β-catenin pathway can be regulated by other additional cues, such as protein interactions, microenvironments, and signaling pathways [28]–[32]. It has been reported that many proteins in the S100 family, such as S100A6, S100B and S100P, negatively regulate cacyBP/SIP-mediated β-catenin degradation [33]–[35], while S100A7 inhibits tumorigenesis via down-regulation of the β-catenin [36]. However, it still remains unclear whether S100A8 and S100A9 also regulate Wnt/β-catenin pathway and thereby exert their effects on CRC progression.

In the present study, the correlation between the expression of S100A8 or S100A9 in CRC cells and the clinicopathological parameters of the patients with CRC was investigated. We found that S100A8 and S100A9 were elevated in CRC, and their expression in tumor cells was associated with the differentiation, Dukes stage and lymph node metastasis. Then the influence of S100A8 and S100A9 in the viability and migration of human colorectal carcinoma cell lines (HCT116 and SW480) and in Wnt/β-catenin pathway was analyzed. We found that the recombinant S100A8 and S100A9 proteins promote the viability and migration of CRC cells and upregulate the activity of Wnt/β-catenin pathway, and that their effect on cell viability and migration was partially mediated by upregulating Wnt/β-catenin pathway. Our study highlights the significance of S100A8 and S100A9 in the progression of CRC, and may provide the potential therapeutic targets in CRC.

## Materials and Methods

### Colorectal tissue samples collection, Ethics Statement, and Cell lines

Colorectal carcinoma and matching distal normal tissues were collected from 42 patients (19 men and 23 women) who had undergone colorectal resection at the First Affiliated Hospital of the Chongqing Medical University. The patients received no chemotherapy, hormonal therapy or radiotherapy before surgery, and the written informed consent was received from all participants. This study was approved by the Ethics Committee of Chongqing Medical University (protocol number 2012-19).

Human colorectal carcinoma cell lines HCT116 and SW480 were purchased from ATCC (American Type Culture Collection, Manassas, VA). Human embryonic kidney cell line 293 (HEK 293) was purchased from China Center for Type Culture Collection (CCTCC). HCT116, SW480 and HEK293 cells were maintained in modified Eagle's medium (MEM) with 10% fetal bovine serum (FBS, Hyclone, USA), in Roswell Park Memorial Institute (RPMI) −1640 with 10% FBS (GIBCO, USA) and in Dulbecco's modified Eagle's medium (DMEM) with 10% FBS (Hyclone, USA), respectively. Cell culture was maintained at 37°C in a humid atmosphere containing 5% CO_2_.

### Histopathology examination

The specimens were fixed in 10% buffered-formalin, embedded in paraffin blocks and were serially sectioned for hematoxylin/eosin (HE) staining. These sections were viewed twice by two experienced blinded pathologists to verify the diagnosis, histological differentiation, and pathological stage. These tumors were staged according to Dukes staging system into stage A (limited to the bowel wall, 2 cases), stage B (extension to muscle layer of the colon or rectum, 13 cases), stage C (regional lymph node metastasis, 21 cases) and stage D (distant metastasis, 6 cases). Tumor histological differentiation was graded into well (3 cases), moderately (29 cases) and poorly (10 cases). The information of patients' tumor size, pathology and stages are presented in Table 1.

**Table 1:** Clinicopathological characteristics of 42 patients with colorectal carcinoma.

Characteristics	n
Gender	
Male	19
Female	23
Age (years)	
≤65	21
>65	21
Location	
Colon	16
Rectum	26
Tumor size (cm)	
<5	23
≥5	19
Histologic differentiation	
Well, Moderately	32
Poorly	10
Dukes stage	
A/B	15
C/D	27
Lymph node metastasis	
Negative	17
Positive	25

The number of samples in each group is shown.

n: number of samples.

### Immunohistochemistry (IHC)

The expression of S100A8 or S100A9 in tissues was examined by IHC. The sections from the formalin fixed, paraffin-embedded tissues were deparaffinized and dehydrated. Then the sections were boiled for 10 min in 0.01 M citrate buffer and incubated with 0.3% hydrogen peroxide (H_2_O_2_) in methanol for 15 min to block endogenous peroxidase. And the sections were incubated with the anti-hS100A8 or anti-hS100A9 monoclonal antibody (1∶300 dilution, Cat#48352 or 58706, respectively, Santa Cruz Biotechnology, Santa Cruz, California, USA) overnight at 4°C, following incubated with secondary antibody tagged with the peroxidase enzyme (Zhongshan Golden Bridge, SP-9002, Beijing, China) for 30 min at room temperature and were visualized with 0.05% 3,3-diaminobenzidine tetrachloride (DAB) until the desired brown reaction product was obtained. The sections were finally counterstained with hematoxylin. The negative control group was carried out with the same steps as described above except replacing the primary antibody with phosphate buffer solution (PBS).

All slides were observed under a Nikon E400 Light Microscope and representative photographs were taken. The immunohistochemical labeling was assessed by two experienced pathologists. The proportion of staining of tumor cells was classified into grade 1 (<10% of positive cells), grade 2 (10–25% of positive cells), grade 3 (26–75% of positive cells) and grade 4 (>75% of positive cells). In addition, its staining intensity was also divided into four subgroups (no staining = 0, weak = 1, moderate = 2, strong = 3). The staining index was obtained by multiplication of proportion and intensity ranging from 0 to 12 [37]. The expression of S100A8 and S100A9 for each section was finally analyzed by a simplified score (score?, staining index 0–1; score?, staining index 2–4; score ?, staining index 6–8; score ?, staining index 9–12). The expression of S100A8 and S100A9 in tumors with a score of at least ? was regards as positive.

### Preparation of the recombinant proteins and amplification of the recombinant adenoviruses

pGST-Moluc-hS100A8 and pGST-Moluc-hS100A9 have been described previously [38]. Briefly, the two plasmids were transformed into *E. coli* (BL21) following the instructions of calcium chloride transformation. Isopropylthio-β-D-galactoside was used to induce the expression of GST-S100A8 and GST-S100A9. After the bacteria were sonicated, the supernatants were collected, spun and incubated with glutathione-Sepharose 4B beads, and GST-S100A8 and GST-S100A9 on the beads were eluted by elution buffer with reduced glutathione. Finally, the proteins were filtered via 0.22 µm membrane and stored at −80°C. The control protein GST was prepared at the same time. Its plasmid is pGST-Moluc.

The recombinant adenovirus carrying β-catenin gene (Adβ-catenin) and β-catenin-siRNA gene (Adsiβ-catenin) and their control (AdGFP or AdRFP) were kindly provided by Professor T.C. He (Medical center, The University of Chicago). All were amplified in HEK293 cells before use [39]–[40].

### Coomassie brilliant blue staining

The recombinant proteins (GST, GST-hS100A8 and GST-hS100A9) were subjected to polyacrylamide gel electrophoresis. Following electrophoresis, the gel was placed in a colloidal coomassie staining solution and incubated for 6 hours to overnight. Then distilled water was used to de-stain the gel until the background was transparent. All steps were done on a rotary shaker with slight mixing.

### Cell viability assay

MTT [3-(4, 5-dimethylthiazol-2-yl)-2, 5-diphe-nyltrazolium bromide] assay was used to assess the cell viability. HCT116 or SW480 cells during log growth stage were seeded in 96-well plates (1000 cells/well) with MEM or RPMI1640 containing 10% FBS and incubated overnight. Then the cells in different groups were treated with and without GST, GST-hS100A8 and GST-hS100A9 in MEM or RPMI-1640 containing 1% FBS for 24, 48, 72, 96 and 120 h, then the MTT reagent (Progema, Madison, WI, USA) was added (20 µl/well) and incubated for 4 h at 37°C, 100 ml dimethyl sulfoxide was added to dissolve formazan product for 10 min at room temperature. Finally, the absorbance was daily measured for the following five days at 492 nm using a microplate reader. Each condition was done in quintuplicate, and the overall experiment was repeated thrice.

### Colony formation assay

HCT116 or SW480 cells during log growth stage were collected and seeded in 6-well plates (100 cells/well), which were treated with and without GST, GST-hS100A8 and GST-hS100A9 at 37°C in a 5% CO_2_ incubator. After two weeks, the cells were stained by crystal violet and clones were counted. The colony-forming rate was obtained as: (colony number/seeded cell number)×100%. The experiment was repeated thrice.

### Transwell migration assay

A chamber of non-type I-collagen-coated 24-well culture inserts (MILLIPORE, USA) was used for the transwell migration assay. Cells (2×10^5^) were seeded in the upper chamber and suspended in 400 µl serum-free MEM or RPMI1640 culture medium in duplicate. The bottom chamber was filled with 500 µl of MEM or RPMI1640 containing 20% FBS as a chemoattractant. After incubation with GST, GST-hS100A8 and GST-hS100A9 for 24 h, the transmembrane cells were dried for 5 min, then fixed with methanol and stained with hematoxylin-eosin for 10min. Finally the transmembrane cells were counted with microscopy at 100×. Mean values were obtained from five randomly selected fields for each well. The experiment was repeated thrice.

### Western blot analysis

For detecting β-catenin, S100A8 and S100A9 in fresh frozen tissues or cells, the tissues and cells were collected and washed with ice-cold PBS, then lysed on ice in a buffer containing 50 mM Tris-HCl, pH 7.5, 100 mM NaCl, and 0.5% Nonidet P-40 and protease inhibitors (Roche Molecular Biochemicals). Nuclear protein and cytoplasmic protein were extracted using Nuclear-Cytosol Extraction Kit (Key GEN, KGP1100, Nanjing, China) according to manufacturer's instructions. Proteins were quantitated by BCA (bicinchoninic acid) assay. The proteins of the lysate were separated in 10% sodium dodecyl sulphate-polyacrylamide gel electrophoresis (SDS-PAGE) and blotted onto the PVDF membranes. Then the membranes were blocked with 5% bovine serum albumin at room temperature for 2 h and incubated with rabbit anti-β-catenin polyclonal antibody (1∶1000 dilution, Cat#7199, Santa Cruz Biotechnology, Santa Cruz, California, USA), or mouse anti-S100A8 monoclonal antibody (1∶1000 dilution, Cat#48352, Santa Cruz Biotechnology, Santa Cruz, California, USA), or mouse anti-S100A9 monoclonal antibody (Santa Cruz, 1∶1000 dilution, Cat#58706, Santa Cruz Biotechnology, Santa Cruz, California, USA), or mouse anti-β-actin monclonal antibody (1∶1000 dilution, Cat#47778, anta Cruz Biotechnology, Santa Cruz, California, USA), or goat anti-Histone antibody (1∶1000 dilution, Cat#8662, anta Cruz Biotechnology, Santa Cruz, California, USA) at 4°C overnight. After washed with TBS supplemented with 0.1% Tween 20 (3×5min), the membranes were incubated with goat anti-rabbit IgG serum (1∶5000 dilution, Zhongshan Golden Bridge, Beijing, China), goat anti-mouse IgG serum (1∶5000 dilution Zhongshan Golden Bridge, Beijing, China) or rabbit anti-goat IgG serum (1∶5000 dilution, Zhongshan Golden Bridge, Beijing, China) for 2 h at room temperature and enhanced chemoluminescence (Millipore Corporation, Billerica, MA, USA) in substrate for 5 to 20 min. The results were recorded by using the Bio-Rad Electrophoresis Documentation (Gel Doc 1000, Bio-Rad, USA) and Quantity One Vesion 4.5.0.

### Immunocytochemistry staining

The cells were plated and cultured onto cleaned-up cover slips. After treated with and without GST (10 µg/ml), GST-hS100A8 (10 µg/ml) and GST-hS100A9 (10 µg/ml) for 48h, the cells were treated with 0.03% H_2_O_2_ for 5 min and incubated with rabbit anti-β-catenin polyclonal antibody (1∶500 dilution, Cat#7199, Santa Cruz Biotechnology, Santa Cruz, California, USA) overnight at 4°C, following incubated for 30 min with secondary antibody tagged with the peroxidase enzyme (Zhongshan Golden Bridge, SP-9001, Beijing, China), and then visualized with 0.05% DAB until the desired brown reaction product was obtained. Finally, all sections were dehydrated, cleared and mounted in a neutral gum under cover slips. The negative control group was carried out with the same steps as described above except replacing the primary antibody with PBS. The staining of samples was observed under an inverted phase contrast microscope and representative images were captured (Olympus B×40, Japan).

### RNA isolation and semi- quantitative RT-PCR

Total RNA was extracted from both fresh frozen tissues and cells using Trizol (Invitrogen, Carlsbad, CA, USA). Semi-quantitative reverse transcriptase polymerase chain reaction (RT-PCR) was carried out as described by using the Takara RNA PCR kit. Primers were also synthesized by Invitrogen. The mRNA levels of S100A8, S100A9, c-myc, and *MMP7* were detected by RT-PCR. GAPDH was used as an endogenous control. The PCR primers (see Table S1) were designed by using the Primer3 program. The PCR products were separated by 1.5% agarose gels and stained with ethidium bromide. The results were recorded by the Gel imaging system (Gel Doc 1000, Bio-Rad, USA) and Quantity One Vesion 4.5.0.

### The influence of β-catenin on S100A8- or S100A9-induced cell viability and migration

To detect the effects of β-catenin on the biological roles of S100A8 and S100A9 in CRC cell lines, the cells were grown and infected with Adsiβ-catenin or Adβ-catenin for 36 h and then treated with and without GST, GST-hS100A8 or GST-hS100A9 subsequent 24 and 72 h. After incubation for the subsequent 24 h, the cell migration capability was analyzed by transwell migration assay, and after 72 h, the cell viability was analyzed by MTT assay. The overall experiment was repeated thrice.

### Statistical analyses

All the values and figures in the text were presented as mean ± SEM (standard error of the mean). One-way ANOVA followed by the S-N-K test was used for the analyses of semi-quantitative RT-PCR, colony formation, cell viability, transwell migration and western blot assay. Chi-square test and Yates-corrected chi-squared test were used to analyze the relationship between the expression of S100A8 or S100A9 in tumors and various clinicopathological parameters. All the statistical analyses were performed using GraphPad Prism 5 (GraphPad Software, La Jolla, California, USA). Significant probability values were indicated as p<0.05*, p<0.01**, p<0.001***.

## Results

### Elevated expression of S100A8 and S100A9 in tumor cells of colorectal carcinoma tissue

**Figure 1:**
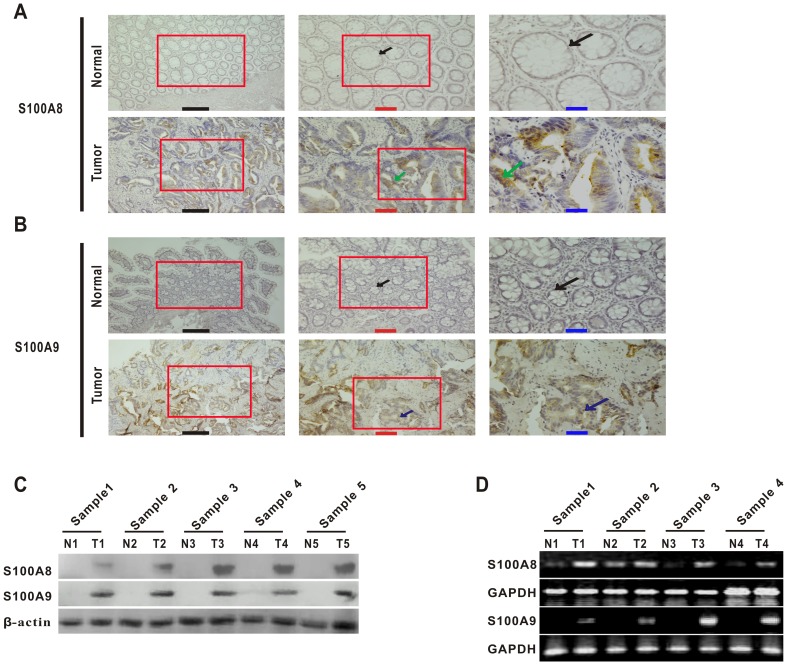
S100A8 and S100A9 are highly expressed in human colorectal carcinoma. (A) Immunohistochemistry for S100A8 in representative samples of tumor tissue and matching distal normal tissue from the same patient (moderate differentiation and Dukes' B): black arrow, normal glandular epithelial cells; green arrow, S100A8-expressing tumor cells (brown). High-magnification images of the red boxed areas are shown in the middle and right. Black scale bars = 500 µm; red scale bars = 200 µm; blue scale bars = 100 µm. (B) Immunocytochemical staining of S100A9 in representative samples of tumor tissue and matching distal normal tissue from the same patient (well differentiation and Dukes' A): black arrow, normal glandular epithelial cells; blue arrow, S100A9-expressing tumor cells (brown). High-magnification images of the red boxed areas are shown in the middle and right. Black scale bars = 500 µm; red scale bars = 200 µm; blue scale bars = 100 µm. (C) Western blot analyses for the expression of S100A8 and S100A9 in colorectal carcinoma and matched distal normal tissues from five randomly selected patients' samples. Sample 1, moderate differentiation and Dukes' A; Sample 2, moderate differentiation and Dukes' A; Sample 3, poor differentiation and Dukes' C; Sample 4, moderate differentiation and Dukes' C; Sample 5, poor differentiation and Dukes' D. T, tumors; N, matching distal normal tissues. β-actin were used as an internal reference control. (D) The transcription levels of S100A8 and S100A9 were analyzed using RT-PCR in four random colorectal carcinoma patients. Sample 1, poor differentiation and Dukes' D; Sample 2, moderate differentiation and Dukes' B; Sample 3, moderate differentiation and Dukes' C; Sample 4, moderate differentiation and Dukes' B. T, tumors; N, matching distal normal tissues. GAPDH was used as an internal reference control.

We performed immunohistochemical (IHC) staining to detect the expression of S100A8 and S100A9 in sections of 42 pairs of samples (CRC and matching distal normal tissues) and found that S100A8 and S100A9 were elevated in tumor cells of CRC tissues compared with the matching distal normal tissues (Fig. 1A and B). This was also confirmed by western blot in five randomly selected patients' samples (Fig. 1C). Furthermore, their mRNA levels were also elevated in tumor tissues from randomly selected patients comparing their matching distal normal tissues (Fig. 1D). The positive rates of S100A8 and S100A9 in tumor cells were 54.8% and 69.1% in the CRC tissues, compared to 7.1% and 16.7% in the matching distal normal tissues respectively (Table 2, p<0.001).

**Table 2:** Expression of S100A8 and S100A9 in paired of colorectal carcinoma and matched distal normal tissues.

		S100A8 expression			S100A9 expression			
		Negative	Positive		Negative	Positive		
		ScoreI	ScoreII, III and IV		ScoreI	ScoreII, III and IV		
Group	n	n (%)	n (%)	*p-Value	n (%)	n (%)	*p-Value	
CRC	42	19 (45.2)	23 (54.8)	p< 0.001	13 (30.9)	29 (69.1)	p< 0.001	
Normal tissues	42	39 (92.9)	3 (7.1)		35 (83.3)	7 (16.7)		

CRC, colorectal carcinoma samples; normal tissue, matching distal normal tissues; n, number of samples. The proportion of staining of tumor cells was classified in four grades (<10% = 1, 10–25% = 2, 26–75% = 3, >75% = 4). In addition, its staining intensity was also divided into four subgroups (no staining = 0, weak = 1, moderate = 2, strong = 3). Staining index (value range, 0–12) = proportion×intensity. The expression of S100A8 and S100A9 was finally analyzed by a simplified score (scoreI, staining index 0–1; scoreII, staining index 2–4; score III, staining index 6–8; score IV, staining index 9–12). The expression of S100A8 and S100A9 in tumors with staining score of at least II were regards as positive. Pearson chi-squared test. *p-value<0.05 was considered significant.

### Association of the expression of S100A8 or S100A9 with the clinicopathological features in CRC

To further investigate whether the expression of S100A8 and S100A9 in tumor cells was associated with disease progression, we analyzed their expression against the clinicopathological parameters, including gender, age, tumor location, tumor size, histological differentiation, Dukes stage and lymph node metastasis (Table 3). Our data showed that expression of S100A8 and S100A9 in the poorly differentiated CRC tissues was higher than that of the well and moderately differentiated CRC tissues (p<0.05 and p<0.05, respectively), and that they were frequently found in the patients with lymph nodes metastasis (p<0.01 and p<0.01, respectively). With regard to Dukes stage, the levels of S100A8 and S100A9 of advanced CRC samples were much higher than that of early stage CRC samples (p<0.01 and p<0.01, respectively). These results suggested that their positive expression in tumor cells was associated with histological grade, Dukes stage and lymph node metastasis, but not with gender, age, tumor location and tumor size.

**Table 3:** Relationship between S100A8 or S100A9 expression and clinicopathological parameters of patients.

		S100A8 expression			S100A9 expression		
		Negative	Positive		Negative	Positive	
		ScoreI	ScoreII, III and IV		ScoreI	ScoreII, III and IV	
Characteristics	n	n (%)	n (%)	*p-Value	n (%)	n (%)	*p-Value
Gender							
Male	19	10 (52.6)	9 (47.4)	p = 0.382^a^	7 (36.8)	12 (63.2)	p = 0.453^a^
Female	23	9 (39.1)	14 (60.9)		6 (26.1)	17 (73.9)	
Age (years)							
≤65	21	12 (57.1)	9 (42.9)	p = 0.121^a^	8 (38.1)	13 (61.9)	p = 0.317^a^
>65	21	7 (33.3)	14 (66.7)		5 (23.8)	16 (76.2)	
Location							
Colon	16	6 (37.5)	10 (62.5)	p = 0.429^a^	6 (37.5)	10 (62.5)	p = 0.707^a^
Rectum	26	13 (50)	13 (50)		7 (26.9)	19 (73.1)	
Tumor size (cm)							
<5	23	13 (56.5)	10 (43.5)	p = 0.106^a^	10 (43.4)	13 (56.6)	p = 0.053^a^
≥5	19	6 (31.6)	13 (68.4)		3 (15.8)	16 (84.2)	
Histological differentiation							
Well/moderately	32	18 (56.3)	14 (43.7)	p = 0.028^b^	13 (40.6)	19 (59.4)	p = 0.042^b^
Poorly	10	1 (10)	9 (90)		0 (0)	10 (100)	
Dukes stage							
A/B	15	11 (73.3)	4 (26.7)	p = 0.006^a^	10 (66.7)	5 (33.3)	p = 0.001^a^
C/D	??	8 (29.6)	19 (70.4)		3 (11.1)	24 (88.9)	
Lymph node metastasis							
Negative	17	12 (70.6)	5 (29.4)	p = 0.006^a^	10 (58.9)	7(41.1)	p = 0.001^a^
Positive	25	7 (28)	18 (72)		3 (12)	22 (88)	

CRC, colorectal carcinoma samples; normal tissues, matching distal normal tissues; n, number of samples. The proportion of staining of tumor cells was classified in four grades (<10% = 1, 10–25% = 2, 26–75% = 3, >75% = 4). In addition, its staining intensity was also divided into four subgroups (no staining = 0, weak = 1, moderate = 2, strong = 3). Staining index (value range, 0–12) = proportion×intensity. The expression of S100A8 and S100A9 was finally analyzed by a simplified score (scoreI, staining index 0–1; scoreII, staining index 2–4; score III, staining index 6–8; score IV, staining index 9–12). The expression of S100A8 and S100A9 in tumors with staining score of at least II were regards as positive. a, Pearson chi-squared test; b, Yates-corrected chi-squared test. *p-value<0.05 was considered significant.

### Recombinant S100A8 and S100A9 proteins promote the viability and migration of CRC cells

Extracellular S100A8 and S100A9 represent danger signals and trigger cellular responses in the dose-dependent manner in tumors. To further evaluate the influence of extracellular S100A8 or S100A9 on the viability and migration of CRC cells, we prepared recombinant proteins GST, GST-hS100A8 and GST-hS100A9 to treat cells, and GST was used as the control for GST-hS100A8 and GST-hS100A9. Their purities were all over 90% (by Quantity One Software after SDS-PAGE, see Fig. S1). GST-hS100A8 or GST-hS100A9 was identified by specific anti-S100A8 or anti-S100A9 antibody (see Fig. S1).

**Figure 2:**
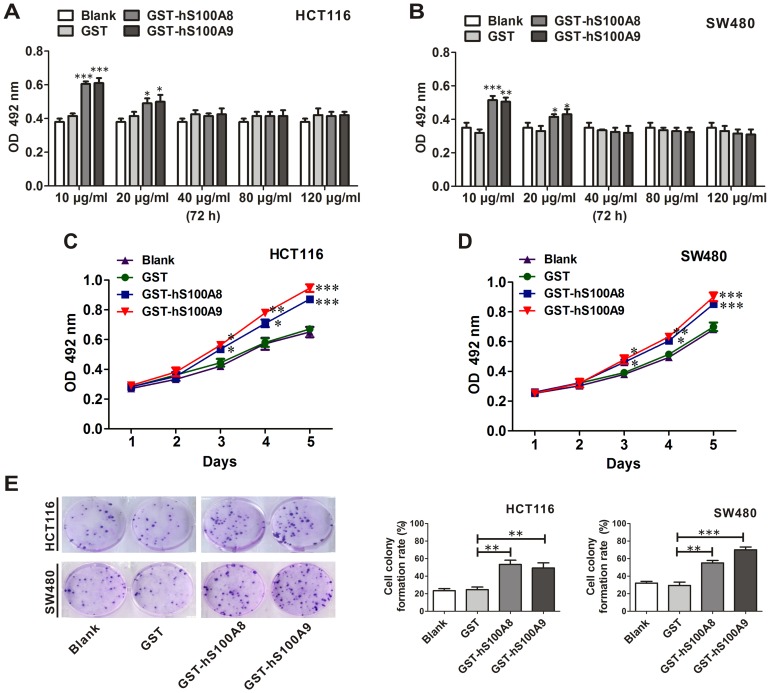
The influence of recombinant S100A8 and S100A9 proteins on the viability of CRC cells. (A) HCT116 and (B) SW480 cells were treated with and without GST, GST-hS100A8 and GST-hS100A9 at different concentrations for 72 h, and cell viability was measured using the MTT assay. Results are expressed as the mean absorbances ± SEM of three independent experiments. * p<0.05, ** p<0.01 and *** p<0.001, all vs. GST control. (C) HCT116 and (D) SW480 cells were treated with and without GST (10 µg/ml), GST-hS100A8 (10 µg/ml) and GST-hS100A9 (10 µg/ml) for continuous five days in medium containing 1% FBS. Cell viability was measured using the MTT assay. Results are expressed as the mean absorbances ± SEM of three independent experiments. * p<0.05, ** p<0.01 and *** p<0.001, all vs. GST control. (E) HCT116 cells and SW480 cells were seeded in a 6-well tissue culture plate, and treated with and without GST (10 µg/ml), GST-hS100A8 (10 µg/ml) or GST-hS100A9 (10 µg/ml) for two weeks for the analysis of colony forming ability. The representative images of the colony-forming units are shown in the left panel, and colony-forming rates for each group are quantified in the middle (HCT116) and right (SW480) panels. The colony-forming rate was obtained as: (colony number/seeded cell number)×100%. The experiment was repeated thrice. ** p<0.01 and *** p<0.001, GST-hS100A8 or GST-hS100A9 vs. GST control.

After HCT116 cells were treated with recombinant GST, GST-hS100A8 or GST-hS100A9 at 10, 20, 40, 80 and 120 µg/ml for 72 h, we found that GST-hS100A8 or GST-hS100A9 proteins at 10 and 20 µg/ml promoted cell viability by using MTT assay (p<0.001 and p<0.05, respectively), while the proteins at 40, 80 and 120 µg/ml had no effect on the cell viability (Fig. 2A). The similar results were also obtained in SW480 cells (Fig. 2B). Furthermore, after treating HCT116 or SW480 cell lines with GST-hS100A8 (10 µg/ml) and GST-hS100A9 (10 µg/ml), the cell viability was increased in a time-dependent manner (Fig. 2C and D). At the same time, after two weeks treatment with GST-hS100A8 (10 µg/ml) or GST-hS100A9 (10 µg/ml), the colony-forming rate of HCT116 cells was increased by 1.20-fold (p<0.01) and 1.04-fold (p<0.01) respectively, compared with GST group (Fig. 2E); the colony-forming rate of SW480 was increased by 0.90-fold (p<0.01) and 1.41-fold (p<0.001) respectively (Fig. 2E).

**Figure 3:**
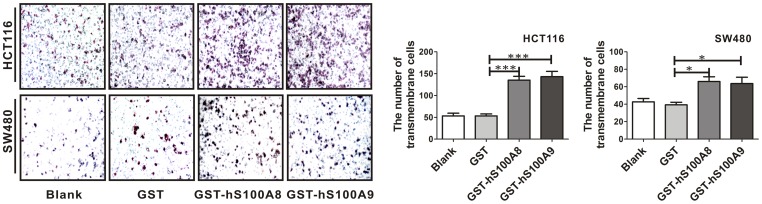
The influence of recombinant S100A8 and S100A9 proteins on the migration of CRC cells. Transwell migration assay was performed on HCT116 and SW480 cells treated with and without GST, GST-hS100A8 or GST-hS100A9 at the concentration of 10 µg/ml. 24 h after treatment, the representative images of transmembrane cells are shown in the left panel, the mean numbers of transmembrane cells ± SEM per microscopic field of three independent experiments are quantified in the middle (HCT116) and right (SW480) panels. Magnification, 100×. * p<0.05 and *** p<0.001, GST-hS100A8 or GST-hS100A9 vs. GST control.

After the HCT116 cells were treated with GST-hS100A8 (10 µg/ml) and GST-hS100A9 (10 µg/ml) for 24 h, the number of migratory cells was significantly increased by 1.53-fold (p<0.001) and 1.67-fold (p<0.001) by transwell migration assay respectively, compared to the cells treated with GST protein (Fig. 3). The similar results were also obtained in SW480 cells, the number of migratory cells was increased by 0.69-fold (p<0.05) and 0.62-fold (p<0.05) respectively (Fig. 3). These data suggested that extracellular S100A8 or S100A9 could promote viability and migration of SW480 and HCT116 cells.

### The effect of S100A8 and S100A9 on activity of Wnt/β-catenin pathway in CRC cells

It is well known that aberrant activation of the canonical Wnt/β-catenin pathway plays a critical role in the development of CRC. To test whether S100A8 and S100A9 can regulate the Wnt/β-catenin pathway, we first investigated the influence of S100A8 and S100A9 on β-catenin level, which is a key effector of this signaling pathway.

**Figure 4:**
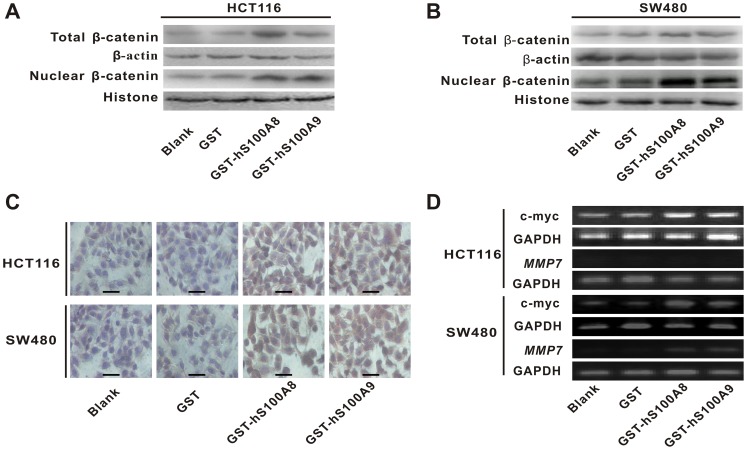
The influence of recombinant S100A8 and S100A9 proteins on the levels of β-catenin and its target gene c-myc and ***MMP7*** **in CRC cells.** (A) HCT116 and (B) SW480 cells were treated with and without GST, GST-hS100A8 or GST-hS100A9 at concentration of 10 µg/ml for 36 h, and total and nuclear β-catenin level were measured by Western blot. β-actin and histone were used as internal reference controls. (C) HCT116 and SW480 cells were treated with and without GST, GST-hS100A8 or GST-hS100A9 at concentration of 10 µg/ml for 48 h, and β-catenin level was analyzed by immunocytochemical (ICC) staining. The representative images are shown in the graph. The intense staining for β-catenin level is in nucleus after treatment with GST-hS100A8 and GST-hS100A9. Black scale bars = 100 µm. (D) HCT116 andSW480 cells were treated with and without GST, GST-hS100A8 or GST-hS100A9 at concentration of 10 µg/ml for 48 h, and the expression of c-myc mRNA and *MMP7* mRNA were detected using RT-PCR. GAPDH was used as an internal reference control.

After HCT116 and SW480 cells were treated with recombinant GST-hS100A8 (10 µg/ml) or GST-hS100A9 (10 µg/ml) for 36 h, we detected β-catenin levels in the whole and nuclear lysates. We found that the levels of total β-catenin and nuclear β-catenin were increased in HCT116 cell line (Fig. 4A). The similar results were also obtained in SW480 cells (Fig. 4B). In addition, immunocytochemistry (ICC) staining confirmed the increase of β-catenin level and showed that the intense staining for β-catenin in nuclei in GST-hS100A8 or GST-hS100A9 group (Fig. 4C). The similar results were also obtained by immune-fluorescence cytochemistry in SW480 cells (see Materials and Methods S1 and Fig. S2). These results suggested that recombinant S100A8 and S100A9 may take part in the Wnt/β-catenin pathway.

To further confirm the influence of recombinant S100A8 and S100A9 proteins on activity of Wnt/β-catenin pathway in CRC cells, RT-PCR was used to detect the transcriptional levels of c-myc and *MMP7*, which are two classic target genes of the pathway [41]–[42]. The result showed that GST-hS100A8 and GST-hS100A9 increased mRNA expression of c-myc in HCT116 cells alone with densitometric semiquantitative assessment of its expression (Fig. S2), while *MMP7* was not detected in the cells (Fig. 4D). At the same time, the two proteins also increased mRNA expression of c-myc and *MMP7* in SW480 cells respectively alone with densitometric semiquantitative assessment of its expression (Fig. 4D and S3). These findings indicated that recombinant S100A8 and S100A9 not only increased total β-catenin level but also promoted the transcription of its target genes c-myc and *MMP7*, which resulted in the upregulation of Wnt/β-catenin pathway.

### Promotion of viability and migration of CRC cells by recombinant S100A8 and S100A9 proteins can be partially mediated by upregulating Wnt/β-catenin pathway

**Figure 5:**
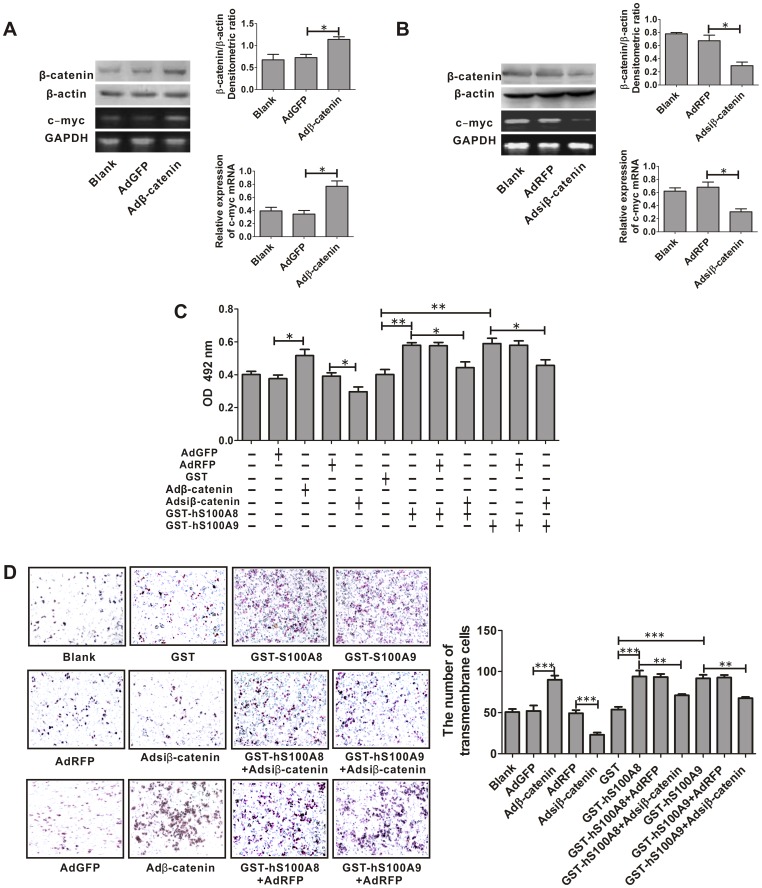
The effect of β-catenin on S100A8- or S100A9- induced viability and migration of SW480 cells. (A) Adβ-catenin-mediated over-expression of β-catenin and its target gene c-myc in SW480 cells. After SW480 cells were infected either with Adβ-catenin or negative control AdGFP for 36 h, β-catenin expression was analyzed by western blot using β-catenin-specific antibody. β-actin was used as internal reference control. The β-catenin/β-actin densitometric ratios are shown in the upper-right panel. The mRNA expression of c-myc was detected using RT-PCR. GAPDH was used as an internal reference control. The relative mRNA expression of c-myc is quantified by c-myc/GAPDH densitometric ratios and is shown in the lower right panel. * p<0.05, Adβ-catenin vs. AdGFP. (B) Adsiβ-catenin-mediated β-catenin gene silencing in SW480 cells. After SW480 cells were infected either with Adsiβ-catenin or negative control AdRFP for 36 h, β-catenin expression was analyzed by western blot using β-catenin-specific antibody. β-actin was used as internal reference control. The β-catenin/β-actin densitometric ratios are shown the upper-right panel. The relative mRNA expression of c-myc is quantified by c-myc/GAPDH densitometric ratios and is shown in the lower right panel. * p<0.05, Adsiβ-catenin vs. AdRFP. (C) After SW480 cells were infected with Adsiβ-catenin or Adβ-catenin for 36 h, then the infectious cells were treated with and without GST (10 µg/ml), GST-hS100A8 (10 µg/ml) or GST-hS100A9 (10 µg/ml) for 72 h and used to analyze cell viability by MTT assay. Absorbance was measured at 492 nm using a microplate reader. Results are expressed as the mean absorbance ± SEM of three independent experiments. Upregulation of β-catenin by infecting with Adβ-catenin increased survival of the SW480 cells, * p<0.05, Adβ-catenin vs. AdGFP; knock-down of β-catenin by infecting with Adsiβ-catenin reduced viability of the SW480 cells and also partially abrogated the promotion of cell viability led by treatment of recombinant S100A8 or S100A9, * p<0.05, Adsiβ-catenin vs. AdRFP; * p<0.05, GST-hS100A8 or GST-hS100A9 vs. GST-hS100A8 or GST-hS100A9+Adsiβ-catenin. (D) After SW480 cells were infected with Adsiβ-catenin or Adβ-catenin for 36 h, the infectious cells were subjected to serum-induced migration by seeding cells in the upper chamber of transwell plates and incubated with and without GST (10 µg/ml), GST-hS100A8 (10 µg/ml) or GST-hS100A9 (10 µg/ml). Transmembrane cells were counted and the representative images are shown in the left panel, and the mean numbers of transmembrane cells ± SEM per microscopic field of three independent experiments are quantified in the right panel. Magnification, 100×. Upregulation of β-catenin by infecting with Adβ-catenin increased migration of the SW480 cells, *** p<0.001, Adβ-catenin vs. AdGFP; knock-down of β-catenin reduced migration of the SW480 cells and also partially abrogated the migration of SW480 cells led by treatment of recombinant S100A8 or S100A9, *** p<0.001, Adsiβ-catenin vs. AdRFP; ** p<0.01, GST-hS100A8 or GST-hS100A9 vs. GST-hS100A8 or GST-hS100A9+Adsiβ-catenin.

As mentioned above, either S100A8 or S100A9 resulted in the accumulation of the β-catenin and upregulation of Wnt/β-catenin pathway in HCT116 or SW480 cells. We next investigated whether elevated β-catenin level was responsible for promotion of cell viability and migration led by GST-hS100A8 or GST-hS100A9. As the promoting effects of recombinant S100A8 and S100A9 on the cell biological behaviors (viability and migration) and β-catenin level of HCT116 and SW480 cell lines were very similar, the subsequent investigations were performed only with SW480 cell line. We transduced the cells with Adβ-catenin and Adsiβ-catenin for over-expression and knock-down of β-catenin level, respectively and after 36 h, β-catenin level was increased by 59.4% and reduced by 56.9%, as compared to the cells infected with AdGFP (control for Adβ-catenin) and AdRFP (control for Adsiβ-catenin), respectively (Fig. 5A and B). At the same time, the level of c-myc mRNA was also increased by 1.1-fold and reduced by 55.8% as compared to the cells infected with AdGFP and AdRFP, respectively (Fig. 5A and B). Then these SW480 cells were treated with and without GST-hS100A8 or GST-hS100A9 for 24 h and 72 h for migration and viability assays respectively. We found that β-catenin over-expression increased cell viability (p<0.05) and on the contrary, β-catenin knock-down reduced cell viability (p<0.05) (Fig. 5C); Furthermore, β-catenin knock-down also partially abolished the cell viability led by treatment of S100A8 (p<0.05) or S100A9 (p<0.05) (Fig. 5C). All suggested that either S100A8- or S100A9-induced promotion of viability of SW480 cells could be partially mediated by elevating β-catenin and upregulating Wnt/β-catenin pathway.

We also found that β-catenin over-expression increased the number of migratory cells (p<0.001), conversely β-catenin knock-down reduced the number of migratory cells (p<0.001). Furthermore, β-catenin knock-down also partially abolished the cell migration led by treatment of S100A8 (p<0.01) or S100A9 (p<0.01) (Fig. 5D). It suggested that S100A8- or S100A9-induced migration of SW480 cells could also be partially mediated by elevating β-catenin and upregulating Wnt/β-catenin pathway.

## Discussion

S100 proteins participate in numerous functions including protein phosphorylation, enzymatic activation, calcium homeostasis, and interaction with cytoskeletal components [43]. Abnormal expressions of S100 proteins, including S100A8 and S100A9, were over-expressed in a variety of different cancers, such as gastric, lung, breast, liver, pancreatic and squamous esophageal carcinomas [7], [44]–[51]. Furthermore, it was reported that S100A8 and S100A9 were detected by using two-dimensional gel electrophoresis showing that there was the increased expression of these two proteins in CRC tissues [10], and that elevated expression of S100A8 and S100A9 was associated with poor differentiation in carcinomas of breast, lung, and thyroid gland [7]–[9]. In the present study, we confirmed the elevation of S100A8 and S100A9 in CRC and found that their elevation in tumor cells was not only associated with histological differentiation but also with Dukes stage and lymph node metastasis in CRC. It suggests that S100A8 and S100A9 may take part in the CRC progression, and may be the potential biomarkers for the histological grade and the metastasis of CRC.

Extracellular S100A8/A9 proteins secreted from cells function as danger associated molecular pattern ligands for cell surface receptors, activating signaling cascades and triggering cellular responses [15]–[21], [52]. For the roles of extracellular S100A8 and S100A9, we used recombinant S100A8 or S100A9 protein (10 µg/ml) to treat CRC cell lines HCT116 and SW480 and found an increase of cell viability and migration. These findings are in agreement with previous data, showing that extracellular S100A8 and S100A9 proteins promoted cell viability and migration in various human cancer cells such as MCF-7, MDA-MB231, SHEP, Kelly, CT-26, PNT1A, HepG2, HUVEC and neutrophil [15], [18]-[21]. In addition, our results showed that higher concentrations of recombinant S100A8 or S100A9 protein monomer (40-120 µg/ml) had no effect on the survival of HCT116 and SW480 cells, which is supported by previous studies showed similar effects on HepG2 cells, mammary carcinoma cells and cervical cancer cells [16], [21], [53]. However, Some previous reports demonstrated that S100A8/A9 heterodimer at the range of 25-250 µg/ml exerted apoptotic effects on certain types of cells, and one possible mechanism is that extracellular chelation of zinc by S100A8/A9 heterodimer decreased the intracellular pool of this ion resulting in the activation of caspase-3 because caspase-3 zymogen (pro-caspase-3) is stabilized in the presence of zinc ions [15]–[17], [54]–[55]. The reason that their monomers at high concentrations had no effect on cell survival was because the monomers had lower specific activity for chelation of zinc than the S100A8/A9 heterodimer [17].

S100 proteins have common structural features and display sequence homology [56]. It has been reported that S100 family members including S100A6, S100A7, S100B and S100P regulate β-catenin degradation [33]-[36]. So we investigated whether S100A8 and S100A9 can regulate Wnt/β-catenin pathway, which is closely related to the progression, metastasis and prognosis of CRC [57]-[60], and found that the recombinant S100A8 and S100A9 proteins could enhance accumulation of β-catenin, and upregulate mRNA expression of c-myc and *MMP7*, the target genes of the pathway, in the cell lines. All suggest that extracellular S100A8 and S100A9 can upregulate Wnt/β-catenin pathway. Based on the previous studies showed that β-catenin upregulation was involved in the proliferation and migration of colon cancer cells [61]-[65], we then explore whether upregulation of Wnt/β-catenin pathway was responsible for S100A8- or S100A9-induced promotion of cell viability and migration, and observed that S100A8- and S100A9-induced survival and migration of CRC cells could be partially mediated by elevating β-catenin and upregulating Wnt/β-catenin pathway.

Extracellular S100A8 and S100A9 in tumor microenvironment may mainly exert their biological roles in CRC progression, and their secretion to tumor microenvironment may be affected by a variety of factors including tumor cells, stromal cells, and other composition of the tumor microenvironment. In addition, there are still some unanswered questions about why S100A8 and S100A9 could exert the promotive effects on activation of Wnt/β-catenin pathway in CRC. We speculate that these promotive effects on Wnt/β-catenin pathway might be via some cell surface receptors. Two pattern recognition receptors, Toll-Like receptor 4 (TLR4) and receptor for advanced glycation end products (RAGE), have shown to be involved in S100A8/A9-mediated pathologic effects such as infection, autoimmunity and cancer [15], [52], [66]-[67]. For example, their interaction with TLR4 increased the systemic autoimmunity and promoted the endotoxin-induced lethality [68]-[69]. RAGE has been proposed to serve as a primary receptor for S100 proteins in the extracellular space, and plays an important role in S100A8- or S100A9-induced proliferation and migration of prostate and breast and colon cancer cells involving the MAPK/NF-κB signaling [18]–[20], [56], [70]–[71]. RAGE has been reported to increase the β-catenin level in human umbilical vein endothelial cell line (ECV–304) [72]. In addition, MAPK pathways have been shown to have unique and provocative roles that impact the Wnt/β-catenin signaling [73]-[76]. Cross-regulation between the Wnt and nuclear factor (NF)-κB signaling pathways was also emerged as an important area for regulating a diverse array of genes and pathways in chronic inflammation and tumor progress [77]. In the future, it is necessary to investigate whether RAGE and its downstream signaling pathways such as MAPK, NF-κB or GSK3β pathways are directly or indirectly involved in the effects of extracellular S100A8 or S100A9 on activation of Wnt/β-catenin pathway.

In conclusion, this data demonstrates that expression of S100A8 and S100A9 in tumor cells is associated with differentiation, Dukes stage and lymph node metastasis in CRC. Furthermore, our data also show that extracellular S100A8 and S100A9 could promote viability and migration of colorectal cancer cell lines HCT116 and SW480 through upregulation of Wnt/β-catenin pathway. Take together, our results provide important information regarding the roles of S100A8 and S100A9 in malignancy and should be paid much attention considering that S100A8 and S100A9 has been increasingly recognized as tumor markers and also as new molecular targets for developing cancer therapeutics.

## Supporting Information

Figure S1 **Identification of GST-S100A8 and GST-S100A9 by SDS-PAGE and Western blotting.** (A) Recombinant human GST-S100A8 was about 37 kDa, GST-S100A9 was about 40 kDa and GST was about 26 kDa; their purities were all >90% (by Quantity One Software after SDS-PAGE). (B) GST-S100A8 and GST-S100A9 was recognized by anti-S100A8 and anti-S100A9 antibodies through western blotting. Left lane 1, GST protein; left lane 2, GST-hS100A8 protein. Right lane 1, GST protein; right lane 2, GST-hS100A9 protein. kDa, kilodalton.
(TIF)

Figure S2 **Immunofluorescence analysis of β-catenin expression in S100A8- and S100A9-treated SW480 cells.** SW480 cells were treated with and without recombinant S100A8 or S100A9 protein (10 µg/ml) for 36 h before being stained by the antibody against β-catenin, and then FITC-labeled secondary antibody were applied (green fluorescence). The nucleus was counterstained with DAPI (blue). The images were visualized under a laser scanning confocal microscope. The representative images are shown in the graph. The intense fluorescence for β-catenin level is in nucleus after treatment with GST-hS100A8 and GST-hS100A9. Red scale bars = 100 µm.
(TIF)

Figure S3 **The effect of recombinant S100A8 and S100A9 proteins on mRNA expression of c-myc and** ***MMP7*** **in CRC cells.** The mRNA expression of c-myc and *MMP7* was detected using RT-PCR. GAPDH was used as an internal reference control. The relative mRNA expression of c-myc or *MMP7* is quantified by c-myc/GAPDH or *MMP7*/GAPDH densitometric ratios and is shown in the graph. * p<0.05, ** p<0.01 and **** p<0.001, all vs. GST control.
(TIF)

Table S1 **Primers used in RT-PCR.**
(DOC)

Materials and Methods S1 (DOC)
