# Correction: S100A8 and S100A9 Are Associated with Colorectal Carcinoma Progression and Contribute to Colorectal Carcinoma Cell Survival and Migration via Wnt/β-Catenin Pathway

**DOI:** 10.1371/annotation/61e0cb2d-6d8c-41d7-99f2-d1b97581e207

**Published:** 2013-05-02

**Authors:** Liang Duan, Rui Wu, Liwei Ye, Haiyan Wang, Xia Yang, Yunyuan Zhang, Xian Chen, Guowei Zuo, Yan Zhang, Yaguang Weng, Jinyong Luo, Min Tang, Qiong Shi, Tongchuan He, Lan Zhou

There was an error in the Funding statement. The correct statement should be:

This work was supported in part by research grants from the Natural Science Foundation of China (#30772548 to Dr. Zhou), and Foundation from Excellent Master Dissertation of College of Laboratory Medicine at Chongqing Medical University (#201202). The funders had no role in study design, data collection and analysis, decision to publish, or preparation of the manuscript. 

