# Supplementary figures and images for: S100A8 and S100A9 Are Associated with Colorectal Carcinoma Progression and Contribute to Colorectal Carcinoma Cell Survival and Migration via Wnt/β-Catenin Pathway
Source: PLoS One. 2013 Apr 26;8(4):e62092. doi: 10.1371/journal.pone.0062092 (PMC3637369; doi:10.1371/journal.pone.0062092)

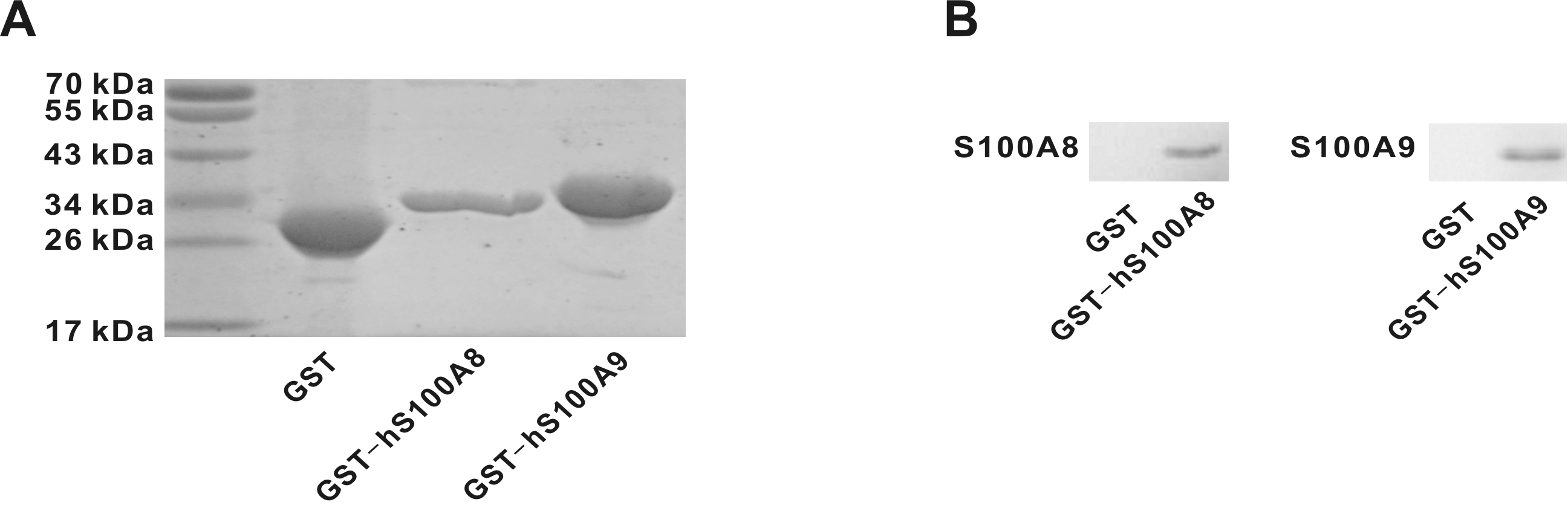

Supplement: Figure S1 — Identification of GST-S100A8 and GST-S100A9 by SDS-PAGE and Western blotting. (A) Recombinant human GST-S100A8 was about 37 kDa, GST-S100A9 was about 40 kDa and GST was about 26 kDa; their purities were all >90% (by Quantity One Software after SDS-PAGE). (B) GST-S100A8 and GST-S100A9 was recognized by anti-S100A8 and anti-S100A9 antibodies through western blotting. Left lane 1, GST protein; left lane 2, GST-hS100A8 protein. Right lane 1, GST protein; right lane 2, GST-hS100A9 protein. kDa, kilodalton. (TIF) [file pone.0062092.s001.tif]

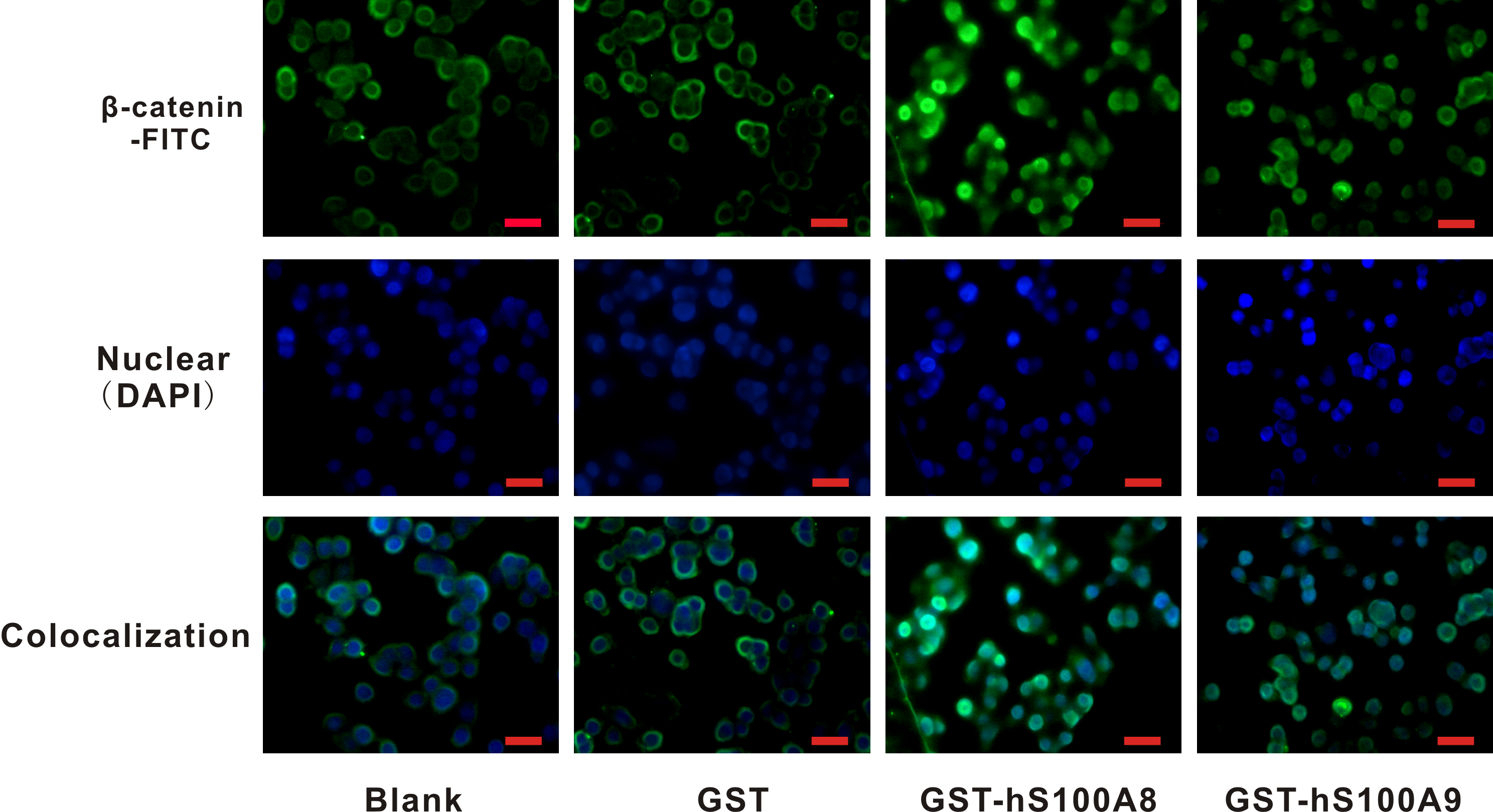

Supplement: Figure S2 — Immunofluorescence analysis of β-catenin expression in S100A8- and S100A9-treated SW480 cells. SW480 cells were treated with and without recombinant S100A8 or S100A9 protein (10 µg/ml) for 36 h before being stained by the antibody against β-catenin, and then FITC-labeled secondary antibody were applied (green fluorescence). The nucleus was counterstained with DAPI (blue). The images were visualized under a laser scanning confocal microscope. The representative images are shown in the graph. The intense fluorescence for β-catenin level is in nucleus after treatment with GST-hS100A8 and GST-hS100A9. Red scale bars = 100 µm. (TIF) [file pone.0062092.s002.tif]

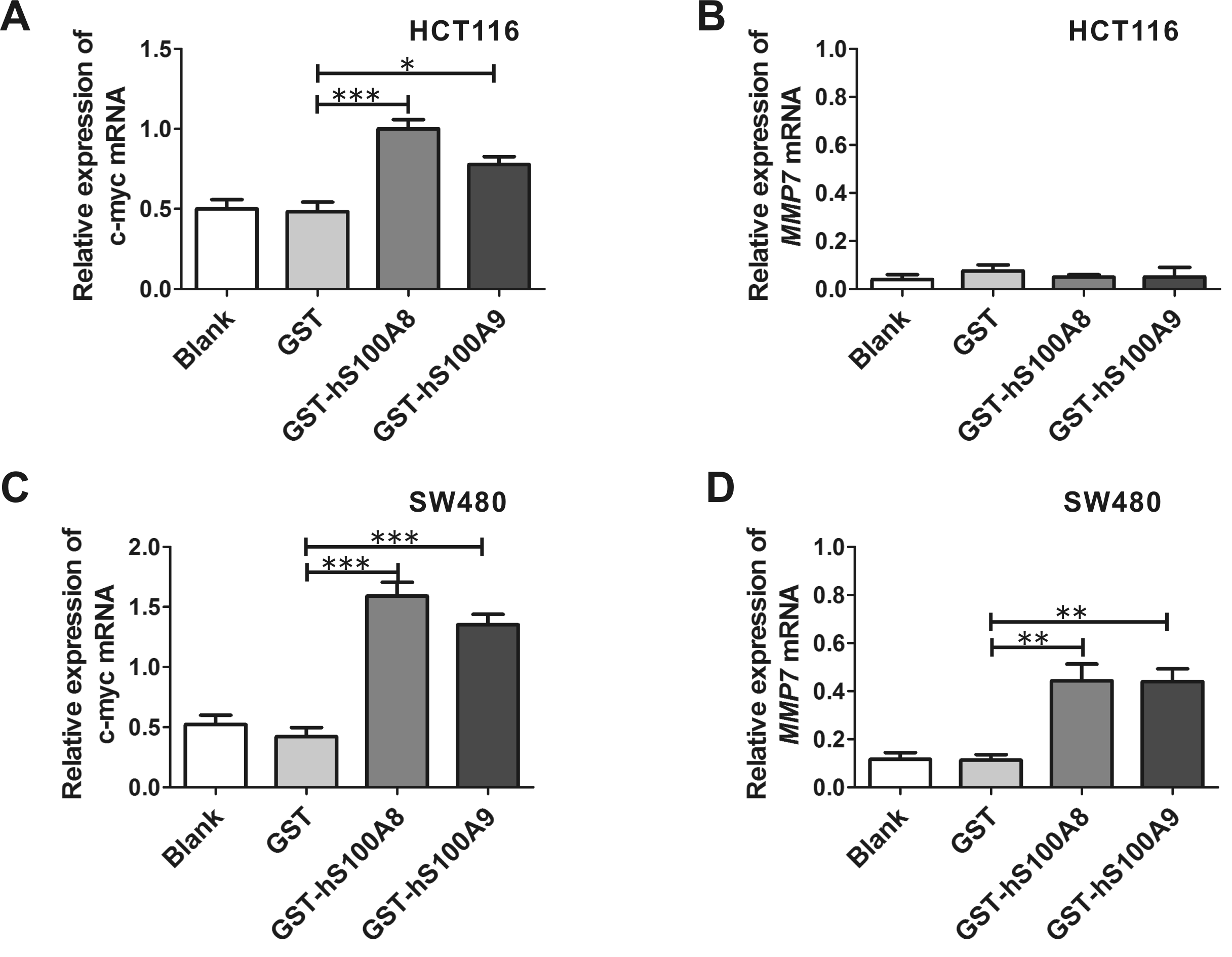

Supplement: Figure S3 — The effect of recombinant S100A8 and S100A9 proteins on mRNA expression of c-myc and MMP7 in CRC cells. The mRNA expression of c-myc and MMP7 was detected using RT-PCR. GAPDH was used as an internal reference control. The relative mRNA expression of c-myc or MMP7 is quantified by c-myc/GAPDH or MMP7/GAPDH densitometric ratios and is shown in the graph. * p<0.05, ** p<0.01 and **** p<0.001, all vs. GST control. (TIF) [file pone.0062092.s003.tif]
