# Supplementary material for: S100A8 and S100A9 Are Associated with Colorectal Carcinoma Progression and Contribute to Colorectal Carcinoma Cell Survival and Migration via Wnt/β-Catenin Pathway
Source: PLoS One. 2013 Apr 26;8(4):e62092. doi: 10.1371/journal.pone.0062092 (PMC3637369; doi:10.1371/journal.pone.0062092)
Supplement: Table S1 — Primers used in RT-PCR. (DOC) [file pone.0062092.s004.doc]

**Table S1 Primers used in RT-PCR**

| **Gene** | **Primer sequences** | |
| --- | --- | --- |
|  | **Forward** | **Reverse** |
| Human S100A8 | 5′-ATTTCCATGCCGTCTACAGG-3′ | 5′-TGGCTTTCTTCATGGCTTTT-3′ |
| Human S100A9 | 5′-TCATCAACACCTTCCACCAA-3′ | 5′-TTAGCCTCGCCATCAGCA-3′ |
| c-myc | 5′-TACCCTCTCAACGACAGCAG-3′ | 5′-TCTTGACATTCTCCTCGGTG-3′ |
| *MMP7* | 5′-GGAGGAGATGCTCACTTCGA-3′ | 5′-AGGAATGTCCCATACCCAAAGA-3′ |
| GAPDH | 5′-CAGCGACACCCACTCCTC-3′ | 5′-TGAGGTCCACCACCCTGT-3′ |
