# Supplementary material for: S100A8 and S100A9 Are Associated with Colorectal Carcinoma Progression and Contribute to Colorectal Carcinoma Cell Survival and Migration via Wnt/β-Catenin Pathway
Source: PLoS One. 2013 Apr 26;8(4):e62092. doi: 10.1371/journal.pone.0062092 (PMC3637369; doi:10.1371/journal.pone.0062092)
Supplement: Materials and Methods S1 — (DOC) [file pone.0062092.s005.doc]

**Materials and Methods S1**

**Immunofluorescence Staining**

The cells were plated and cultured onto cleaned-up cover slips. After treated with and without GST (10 μg/ml), GST-hS100A8 (10 μg/ml) and GST-hS100A9 (10 μg/ml) for 48h, the cells were washed with PBS and fixed in 4% paraformaldehyde, then permeabilized with 0.2% Triton X-100. Cover slips were rinsed and incubated with blocking serum (goat serum) for 15min at 37°C and then incubated with primary rabbit anti-β-catenin polyclonal antibody (1:50 dilution, Cat#47778, anta Cruz Biotechnology, Santa Cruz, California, USA) overnight at 4°C. After three washes with PBS, the cells were stained with the corresponding FITC-conjugated secondary antibodies (1:100 dilution, Cat#L1709, Santa Cruz Biotechnology, Santa Cruz, California, USA). To visualize nuclei, cells were stained with 10 μ g/ml DAPI. The fluorescent images were then observed and analyzed using a laser scanning confocal microscope.
